# Caregiving Concerns in Lewy Body Dementia

**DOI:** 10.1002/alz.087840

**Published:** 2025-01-09

**Authors:** Ece Bayram, Bhavana Patel, Kelly E Stacy, Morgan Farley, Noheli Bedenfield, Angela Taylor, Julia M Wood, Melissa J Armstrong, Samantha K. Holden, Lewy Body Dementia Association Community Engagement Group

**Affiliations:** ^1^ University of California San Diego, La Jolla, CA USA; ^2^ University of Florida, Gainesville, FL USA; ^3^ University of Cincinnati College of Nursing, Cincinnati, OH USA; ^4^ University of Colorado School of Medicine, Aurora, CO USA; ^5^ Lewy Body Dementia Association, Lilburn, GA USA

## Abstract

**Background:**

Lewy body dementia (LBD) is a common and burdensome neurodegenerative dementia, that remains under‐recognized and under‐studied. Caregivers play an important role, although research on their needs is lacking. Understanding and addressing their concerns can help improve their well‐being, the care they provide for their loved ones, and also participation in research.

**Methods:**

Between April 7, 2021, and July 1, 2021, we queried the research priorities, symptom burden, gaps in care, and concerns of people with LBD, and caregivers of people with LBD through forced ranking in an anonymous 15‐20 minute web‐based survey. It was designed by the LBD Association Research Centers of Excellence Community Engagement Working Group and distributed by the LBD Association.

**Results:**

Participants included 832 caregivers (550 current, 280 former, 2 did not specify) with the majority caring for their spouse/partner (63%). They were most likely to be 70‐79 years old (33.5%), married/in a domestic partnership (71.3%), have a bachelor’s or graduate degree (32%, 34.2%), identify as woman (84%), and White (93.4%). Current caregivers were caring for a person who had been diagnosed within the past year (28.4%). Formal caregivers reported their loved one most likely died within the past year (37.5%). Overall, 80% had not participated in research previously. They were frequently concerned about how to manage caregiving alongside other responsibilities (50.1% on a daily basis), what to expect with disease progression (49.7% on a daily basis), how to handle behavior changes (45.9%), and how to support the independence of their loved one (42.6%). They were least concerned about how to obtain medical care for themselves; 34.9% never experienced this concern.

**Conclusions:**

Caregivers of people with LBD experience multiple concerns on a daily basis. Providing support, creating resources, and raising awareness about the available resources can benefit both the caregivers and their loved ones with LBD. Although our cohort was not diverse and the results should not be generalized, our findings can guide future efforts to include caregivers in clinical care, education, and research. Caregivers can provide invaluable information regarding their loved one’s state, the effectiveness of treatments, and the feasibility of research studies.

